# Pathology of Muscæ Volitantes

**Published:** 1829-04-01

**Authors:** 


### XXIV.

### Pathology of Muscæ Volitantes. By Dr. Parfait Landraw.

The following fact is so very curious, that, had the statement not been verified by three or four respectable physicians and surgeons, we should have been inclined to view it as possessing the same kind of authenticity as that which is attached to Mr. Liston's lectures on aneurism, in the Lancet.

On the 17th August, M. Gally, physician of the Perigueux Hospital, brought to the author of the case, M. Audebert, an old magistrate, aged 70 years, who complained of having a derangement in the sight of his right eye, which gave him much inconvenience for some years past: namely, muscæ volitantes, black specks, and other images assuming various forms.

On examination, the pupils were found to be rather contracted; and, when Dr. P. looked attentively into the posterior chamber of the right eye, Dr. P. could perceive numerous small bodies floating about in the posterior chamber of the eye, glittering with a kind of phosphoric brilliancy. The Doctor at first distrusted the evidence of his own senses, and proposed a dilatation of the pupils, by means of belladonna, which was effected, and then M. Gally and himself, together with M. Renaud, surgeon to the hospital, and Dr. Vidal, had a full opportunity of clearly observing the corpuscles above mentioned floating about in the posterior chamber of the eye, when the eye was in motion, and settling down, so as almost entirely to disappear, when the organ was quiescent. These phenomena could be distinguished by the naked eye; but, to remove ambiguity, a lens of some power was employed, and the same facts were verified.

We need hardly remark, that muscæ volitantes have been hitherto accounted for in a manner very different from the above. They have been attributed to a particular condition of the internal membranes of the eye—to varicose vessels expanded on these membranes, or in the humours of the eye—and to an affection of the retina. Demours, however, has observed that these muscæ volitantes arise and subside, according as the eye is in motion or at rest—a fact which accords with the phenomena that appeared in the present case.

There is one difficulty in supposing that these bodies are located in the vitreous humour of the eye—namely, the cellular structure of that humour. Our author endeavours to get over this difficulty, by supposing that the cellular texture of the humour is broken down before the muscæ volitantes take place. There is no doubt that the vitreous humour is occasionally found so fluid as to escape in operations on the eye. The case is very curious, and should attract the attention of oculists and others to the subject. The occurrence of muscæ volitantes is very common, and, therefore, opportunities for observation will not be rare. —RÉVUE MED.

